# Automated, electronic monitoring for early detection of sepsis

**DOI:** 10.1186/cc12457

**Published:** 2013-03-19

**Authors:** S Rauch, M Fischer, C Martin, A Sablotzki

**Affiliations:** 1 Alb Fils Kliniken, Göppingen, Germany; 2 Fa. Löser, Leipzig, Germany; 3 Klinikum Sankt Georg, Leipzig, Germany

## Introduction

Early detection of sepsis is important for a sufficient treatment to reduce mortality. We hypothesized that using modified systemic inflammatory response syndrome criteria over 1 hour using an electronic software program facilitates the clinical diagnosis of sepsis.

## Methods

After IRB approval and informed consent we enrolled in this prospective, observational, single-center study 1,119 consecutive patients (age 68.6 ± 16.4, female/male 476/649) admitted over a 6-month period to a surgical ICU. A total 149 of them met modified systemic inflammatory response criteria. Patients were monitored by an electronic software program using live data from the laboratory and bedside monitors to detect modified systemic inflammatory response syndrome criteria persisting over 1 hour. The physicians were blinded to the software program alerts that notified in real time when modified systemic inflammatory response syndrome criteria were detected and persisted over 1 hour, but did not provide treatment recommendations.

## Results

There was a total of 149 modified systemic inflammatory response syndrome criteria alerts. Seventy-four were confirmed as true sepsis cases by physicians. The overall incidence of sepsis was 7%. Patients were categorized into length of stay <24 hours, 24 to 96 hours and >96 hours. The overall sensitivity of our system for detecting sepsis was 68% and the specificity was 91%. The positive predictive value is 34% and the negative predictive value is 98%.

## Conclusion

Real-time alerts using an automated, electronic monitoring of modified systemic inflammatory response syndrome criteria facilitate the clinical diagnosis of sepsis.

